# On the causes of Arctic sea ice in the warm Early Pliocene

**DOI:** 10.1038/s41598-018-37047-y

**Published:** 2019-01-30

**Authors:** Caroline Clotten, Ruediger Stein, Kirsten Fahl, Michael Schreck, Bjørg Risebrobakken, Stijn De Schepper

**Affiliations:** 1 Uni Research Climate, Bjerknes Centre for Climate Research, Jahnebakken 5, 5007 Bergen, Norway; 2 Alfred Wegener Institute Helmholtz Centre for Polar and Marine Research, Am Alten Hafen 26, 27568 Bremerhaven, Germany; 3 MARUM and Faculty of Geosciences, University of Bremen, PO Box 330440, 28334 Bremen, Germany; 4 Department of Geosciences, UiT The Arctic University of Norway in Tromsø, P.O. Box 6050, Langnes, 9037 Tromsø Norway; 5 NORCE Norwegian Research Centre, Bjerknes Centre for Climate Research, Bergen, Norway

## Abstract

Scattered and indirect evidence suggests that sea ice occurred as far south as the Iceland Sea during the Early Pliocene, when the global climate was warmer than present. However, conclusive evidence as well as potential mechanisms governing sea ice occurrence outside the Arctic Ocean during a time with elevated greenhouse gas concentrations are still elusive. Here we present a suite of organic biomarkers and palynological records from the Iceland Sea and Yermak Plateau. We show that sea ice appeared as early as ~4.5 Ma in the Iceland Sea. The sea ice either occurred seasonally or was transported southward with the East Greenland Current. The Yermak Plateau mostly remained free of sea ice and was influenced dominantly by Atlantic water. From ~4.0 Ma, occurrence of extended sea ice conditions at both the Yermak Plateau and Iceland Sea document a substantial expansion of sea ice in the Arctic. The expansion occurred contemporaneous with increased northward heat and moisture transport in the North Atlantic region, which likely led to a fresher Arctic Ocean that favors sea ice formation. This extensive sea ice cover along the pathway of the East Greenland Current gradually isolated Greenland from warmer Atlantic water in the Late Pliocene, providing a positive feedback for ice sheet expansion in Greenland.

## Introduction

Today, Arctic sea ice plays an important role in both regional and global climate due to its effect on Earth’s albedo, ocean-atmosphere exchange and primary productivity^1,2^. As a result of increasing anthropogenic greenhouse gas emissions, Arctic sea ice extent has been declining drastically over the past decades^3^ and the Arctic may become sea ice-free during summer within a few decades^4,5^. Nevertheless, some studies suggest that Arctic sea ice has occurred repeatedly during times with elevated atmospheric CO_2_ concentrations^6^ such as the Middle Eocene^7–9^, the Late Miocene^10^ and the Pliocene^11^. There remains a debate on the existence of perennial or seasonal sea ice in the Late Miocene Arctic Ocean^10,12^, but seasonal sea ice did occur in the marginal Arctic Ocean during the Early Pliocene from ~4.0 Ma^11^. While circumstantial evidence from Early Pliocene palynological and biogenic opal records suggest that sea ice might have occurred in the Iceland Sea^13,14^ and Labrador Sea^15^, a recent biomarker study demonstrates that seasonal sea ice appeared north of Iceland during the Late Pliocene (<3.6 Ma)^16^.

**Figure 1:**
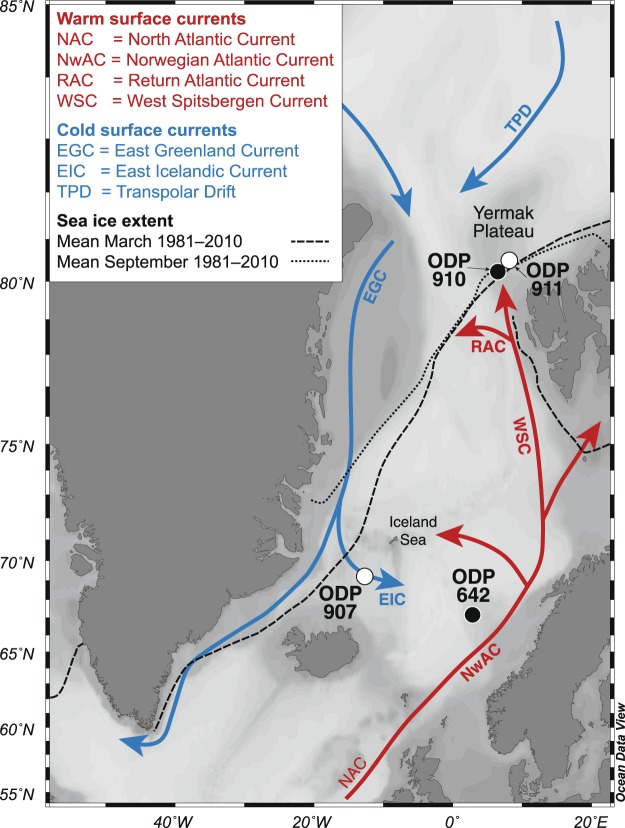
Map of the Nordic Seas and marginal Arctic Ocean showing the modern oceanography and sea ice extent. ODP Site 907 (69°14.989′N, 12°41.894′W; ~1800 m water depth), ODP Hole 911A (80°28.466′N, 8°13.640′E; 902 m water depth) and other core locations discussed in the text (ODP holes 642B and 910C) are indicated. The mean minimum (=September, dotted line) and mean maximum (=March, dashed line) sea ice extent between 1981 and 2010 are shown (from http://nsidc.org/data/G02135)^53^. Map was generated with Ocean Data View^54^.

In the Early Pliocene, when global atmospheric CO_2_ was in the range of 380–400 ppm^17,18^ and the Fram Strait was the only Arctic–Atlantic ocean gateway (the Canadian Arctic Archipelago or CAA^19^ and Barents Sea were sub-aerially exposed^20^), various mechanisms may have caused sea ice occurrence in the Iceland Sea. One important candidate causing sea ice to occur in the Iceland Sea is the East Greenland Current (EGC), which is today the main exporter of sea ice laden, lower salinity Arctic waters from the Arctic Ocean into the Nordic Seas (Fig. 1). This surface current emerged in the Early Pliocene around 4.5 Ma^14^, possibly bringing cool, fresher Arctic water and sea ice southward along the East Greenland coast into the Iceland Sea. Another mechanism for increasing sea ice formation in the Arctic is presented in the theory of ref.^21^, which links the Early Pliocene closure/shoaling of the Central American Seaway (CAS; ~4.5 Ma) to intensified glaciations in the Northern Hemisphere. While the current consensus is that the CAS closure/shoaling was not a direct trigger for the glaciation in the Northern Hemisphere around 2.7 Ma (e.g. ref.^22^), the proposed mechanism predicts the formation of sea ice in the Pliocene Arctic Ocean following enhanced northward heat and moisture supply in the North Atlantic region. This atmospheric moisture is transported via the westerlies to Eurasia, ensuring precipitation that feeds the northward draining Siberian rivers. These rivers then deliver more fresh water to the Arctic Ocean and thereby facilitate sea ice formation. Crucial evidence supporting freshening of the surface waters in the Russian Arctic is still lacking, but paleoclimatic records from the North Atlantic^23^ and Norwegian Sea^24^ indicate increased heat transport and the first seasonal sea ice in the marginal Arctic Ocean (Yermak Plateau) around 4.0 Ma^11^.

Given the current trajectory towards a globally warmer climate and amplified Arctic climate change, it is crucial to document the occurrence of Arctic sea ice during warmer-than-present conditions and understand the underlying mechanisms. Despite not being a perfect analogue for the future climate of our planet^25^, the Early Pliocene does provide the opportunity to study the mechanisms governing sea ice presence in the Iceland Sea in a world characterized by high CO_2_ levels^17^ and global temperatures^26^. Here, we present the first Early Pliocene (~4.9–3.5 Ma) sea ice reconstructions based on the sea ice proxy IP_25_, sterols and palynology from the Iceland Sea Ocean Drilling Program (ODP) Site 907 and Yermak Plateau ODP Hole 911A (Fig. 1) to determine and understand the underlying causes of (seasonal) sea ice presence in the Early Pliocene Arctic.

## Dominantly Ice Free Conditions in the Earliest Pliocene Nordic Seas

**Figure 2:**
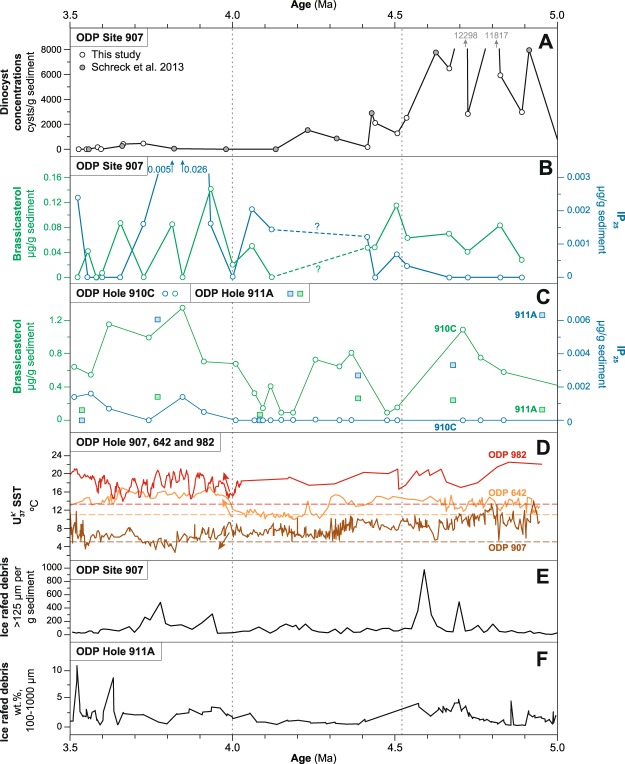
Biomarker and sedimentological data from the Iceland Sea and Yermak Plateau. (**A**) Dinoflagellate cyst concentrations from ODP Site 907 from this study (white circles) and ref.^13^ (gray circles). (**B**) IP_25_ concentrations (blue circles) and brassicasterol concentrations (green circles) from ODP Site 907. (**C**) IP_25_ concentrations (blue circles) and brassicasterol concentrations (green circles) from ODP Hole 910C^11^. Blue squares indicate IP_25_ concentrations and green squares indicate brassicasterol concentrations from ODP Hole 911A (this study). (**D**) High-resolution alkenone-based SST reconstruction from ODP sites 907^29^, 642^24^ and 982^23,29^. Modern summer SSTs for each location is from the World Ocean Atlas 2013 (ref.^28^). (**E**) IRD record from ODP Site 907 from ref.^38^, placed on the ATNTS2012 timescale. (**F**) IRD record from ODP Hole 911A ref.^55^. Vertical dotted lines indicate time slices discussed in the text and used in the reconstructions in Fig. 3.

Our reconstructions reveal sea ice-free conditions and relatively high marine productivity in the Iceland Sea between 5.0 and 4.6 Ma, evidenced by the absence of IP_25_ and relatively high concentrations of the open water biomarker brassicasterol (Fig. 2B). Dinoflagellate cyst concentrations are high (~3,000 to ~12,000 cysts/g; Fig. 2A), and the assemblage has clear Atlantic water characteristics^14^, suggesting a Nordic Seas circulation different from today^13,14,27^. Ice-free conditions are supported by reconstructed summer SSTs ~5 °C higher than modern (Fig. 2D)^28,29^ and high phytoplankton productivity related to the presence of Atlantic water masses in the Iceland Sea.

**Figure 3:**
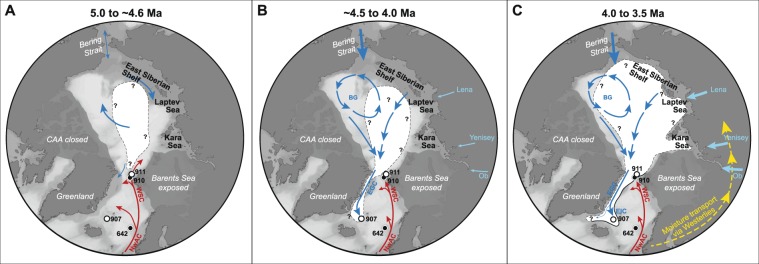
Schematic illustration of sea ice extent in the Arctic Ocean and Iceland Sea during the Pliocene. Warm water surface currents (red): NwAC = Norwegian Atlantic Current, WSC = West Spitsbergen Current. Cool water surface currents (blue): BG = Beaufort gyre, EGC = East Greenland Current, EIC = East Icelandic Current. (**A**) Sea ice (white shaded area) was most likely restricted to the central Arctic Ocean between 5.0 and ~4.6 Ma (e.g. ref.^11^), while Atlantic waters extended into the Iceland Sea (Site 907). (**B**) Seasonal sea ice or sea ice transported with the EGC first appear in the Iceland Sea around 4.5 Ma. The Yermak Plateau was sea ice free^11^, although occasional sea ice may have occurred. (**C**) Sea ice expanded across the Arctic Ocean^56,57^ and established a seasonal/extended sea ice cover on the Yermak Plateau and an extended sea ice cover or possibly a sea ice edge (solid line at edge of white shaded area) in the Iceland Sea. This occurred contemporaneous with increased northward heat transport in the eastern North Atlantic and Norwegian Sea. The northward heat transport also brought moisture that was transported via the westerlies (yellow arrows) towards Eurasia where increased freshwater outflow through the Eurasian rivers (light blue arrows) led to freshening of the Arctic Ocean, which favors sea ice formation. Maps were generated with Ocean Data View^54^.

The low-resolution Yermak Plateau biomarker record suggests occasional sea ice edge conditions at 4.9 and 4.6 Ma (Supplementary Fig. 1B). High IP_25_ and low brassicasterol concentrations indicate seasonal sea ice occurrence at ODP Hole 911A (Fig. 2D), while the absence of IP_25_ and high brassicasterol concentrations at the neighboring ODP Hole 910C indicate sea ice-free conditions and enhanced primary productivity at this time^11^. Yet, a clear, uniform interpretation of the low-resolution biomarker data from these sites, located very close to each other, is difficult to make. The difference in biomarker signature at the two sites could be consistent with a highly variable sea ice margin, comparable to modern conditions in this region (Supplementary Fig. 2)^30^. High brassicasterol concentrations at ODP Hole 910C suggests substantial primary productivity, which is often elevated close to marginal ice zones^10,31^. However, SSTs remain relatively high and indicate an Atlantic rather than polar water influence^11^. Alternatively, as seasonal sea ice was already present in the Arctic Ocean since the Late Miocene^10^, sea ice could have also been exported from the Arctic Ocean towards the Yermak Plateau until it encountered the warmer Atlantic waters of the West Spitsbergen Current. With a sub-aerially exposed Barents Sea^20^, the heat advection through the West Spitsbergen Current towards the Yermak Plateau is increased^32,33^ (Fig. 3A), thereby inhibiting a long-term sea ice cover but allowing occasional local sea ice formation or sea ice export from the Arctic. It is, however, very likely that the different paleoceanographic interpretations for ODP holes 910C and 911A are best explained by the different ages of the investigated samples at both sites and that sea ice appeared in the region only sporadically.

## First Early Pliocene Sea Ice in the Iceland Sea

Sea ice emerged in the Iceland Sea around 4.5 Ma as evidenced by the first occurrence of IP_25_ at ODP Site 907. Together with elevated brassicasterol concentrations (Fig. 2B), the IP_25_ data suggest sea ice edge conditions (Supplementary Fig. 1A), with generally high productivity^10^. Dinoflagellate cyst concentrations are still relatively high for an open ocean site, although lower compared to the preceding interval (Fig. 2A). Several dinoflagellate cyst species disappeared at this site around 4.5 Ma^13,14^, which has been related to the emergence of a modern-like EGC^14^ and the replacement of Atlantic water with cooler, fresher arctic-sourced water. When sea ice occurred at 4.5 Ma, reconstructed summer SSTs of ~10 °C remained well above modern values (Fig. 2D), suggesting either a strong seasonal contrast between warm summers and winters with sea ice, or that transported sea ice melted in the Iceland Sea. While our data unequivocally demonstrates sea ice presence in the Iceland Sea at 4.5 Ma, we cannot conclude whether sea ice formed locally in the Iceland Sea, was exported from the Arctic to the Iceland Sea with the EGC or was a combination of both. In ODP Hole 910C, a significant drop in brassicasterol concentrations (to values below 2 ng/g sediment) and the absence of IP_25_ (Fig. 2C) could be indicative for a permanent sea ice cover at 4.5 Ma (Supplementary Fig. 1B), but the Yermak Plateau most likely still remained sea ice free and dominated by Atlantic water^11^.

Between 4.4 and 4.1 Ma, samples for biomarker analyses were not available at ODP Site 907 due to a heavily sampled sediment core. However, earlier studies show a decrease in summer SSTs from 10 to 6 °C^29^ around 4.4–4.3 Ma and a fragmentary dinoflagellate cyst record with low concentrations to even barren samples^13,14^ (Fig. 2A,D). This suggests a strengthened influence of cool, Arctic waters via the EGC in the Iceland Sea about 100–200 ka later than the first sea ice occurrence at 4.5 Ma. Although this lag is not fully understood, it may be reflecting a gradually intensifying EGC that leads to long-term, cool conditions in the Iceland Sea. The Yermak Plateau generally remained sea ice free^11^, but the occurrence of IP_25_ in a single sample (~4.4 Ma) in ODP Hole 911A reflects that conditions were sporadically suitable for sea ice to be present (Fig. 2C).

Thus, the sea ice reconstructions for the Iceland Sea and the Yermak Plateau around 4.5 Ma correspond favorably to the reorganization of Nordic Seas surface circulation. This paleoceanographic change most likely occurred as a consequence of changed flow direction through the Bering Strait^14^ allowing cool and low salinity Pacific water to enter the Arctic Ocean^15,34–36^. While today, Arctic–Atlantic surface water exchange occurs through both the Fram Strait and the CAA^37^, the latter was closed during the Early Pliocene^19^. Such setting favors the role of the EGC as the only pathway for cool, fresher water and possibly also as sea ice exporter from the Arctic already in the Early Pliocene (Fig. 3B).

## Warming in the Norwegian Sea promotes Arctic Sea Ice Formation

An extended sea ice cover (high IP_25_ and low brassicasterol) alternated with ice edge conditions (high IP_25_ and high brassicasterol) in the Iceland Sea between 4.0 and 3.7 Ma (Fig. 2B; Supplementary Fig. 1A). Dinoflagellate cyst samples are either barren or show very low concentrations during this interval indicating limited productivity (Fig. 2A)^13,14^ and hence possibly harsh conditions due to sea ice presence and/orlow SSTs. Indeed, Iceland Sea summer SSTs decreased further from ~8 to 3 °C^29^ within this interval (Fig. 2D), yet summers largely remained sea ice-free because alkenone production still occurred. At Yermak Plateau ODP Hole 910C, seasonal sea ice appeared shortly after 4.0 Ma^11^, while at ODP Hole 911A, a single sample around 3.8 Ma with high IP_25_ and low brassicasterol concentrations suggests an extended sea ice cover. One sample in ODP Hole 911A could indicate a permanent sea ice cover around 3.5 Ma (Fig. 2C, Supplementary Fig. 1B), but taking the data from ODP Hole 910C into account, a seasonally sea ice covered Yermak Plateau seems more likely.

Around 4.0 Ma, rapid ocean surface warming in the Norwegian Sea (~7 °C in 40 ka; Fig. 2D)^24^, cooler SSTs in the Iceland Sea^29^ and a sea ice cover stretching from the Arctic Ocean to the Iceland Sea all occurred at the same time and established the characteristic zonal surface water gradient of the Nordic Seas. Together, these observations support two major parts of the theory proposed by ref.^21^, namely the northward heat (and associated moisture) transport in the North Atlantic and the enhanced formation of sea ice in the Arctic. Essential for enhanced sea ice formation is a freshening of the Arctic Ocean. It was proposed that the moisture source for this freshening originates from increased northward heat and moisture transport in the North Atlantic^21^. This is evident in the reconstructed high SSTs in the eastern North Atlantic^23^ and Norwegian Sea^24^ around 4.0 Ma (Fig. 2D). There is currently no corroborating data that atmospheric moisture was consequently transported via the westerlies from the Atlantic region to the Eurasian continent where it fed northward-draining rivers, which ultimately freshened the Arctic Ocean to promote sea ice formation. However, our new data do provide evidence that shortly after the rapid temperature increase in the eastern North Atlantic and Norwegian Sea, a major expansion of sea ice cover occurred in the Arctic and extended to the Fram Strait and the Iceland Sea (Figs 2B, 2C and 3C). Further testing of this theory will require gathering data that documents the moisture transport to Siberia, the freshening of the Arctic Ocean via Siberian rivers and consequent sea ice formation.

## Influence of Sea Ice Expansion on Pliocene Arctic Climate

Despite the relatively high Early Pliocene atmospheric CO_2_ concentrations of 380–400 ppm^17,18^, the changes in North Atlantic and Arctic paleoceanography caused sea ice to occur as far south as the Iceland Sea at 4.5 Ma. This development was likely controlled by the emergence of a modern-like EGC, which was established as a consequence of a surface water flow reversal across the Bering Strait^14^. The changed paleoceanography was crucial to allow the import of fresher, cooler Arctic water and sea ice into the Iceland Sea. Whether sea ice was exported directly from the Arctic, or the fresher, cooler water favored local sea ice formation in the Iceland Sea yet remains ambiguous. Nevertheless, the effects of a changed EGC and appearance of sea ice along its pathway are also recognized from the dinoflagellate cyst turnover in the Iceland Sea^14^ and the onset of biosiliceous sedimentation in the Labrador Sea^15^ at 4.5 Ma. Around that time, small IRD amounts are recorded at ODP Site 907, indicating that Greenland did have ice caps or small ice sheets that could produce icebergs and ice rafted detritus (IRD)^38^, albeit in volumes considerably lower than in the Late Pliocene and Quaternary. Tectonic uplift made elevated plateaus available in Greenland during the Early Pliocene^39^ where glaciers and ice caps could nucleate and eventually expand into a large, IRD producing ice sheet. Such large ice sheet started to deliver considerable IRD into the North Atlantic during the Late Pliocene^40^, when the zonal gradient in the Nordic Seas was already established. In fact, the cooler water and more substantial sea ice presence in the Iceland Sea after 4.0 Ma and in the Late Pliocene^16^, may have contributed to the gradual expansion of continental ice in Greenland. A more substantial and long-term sea ice presence along the East Greenland coast acts to thermally isolate Greenland from relatively warmer Atlantic waters^41^ and reduces heat advection^42^, as well as providing a sea ice-albedo feedback and inhibiting ocean–atmosphere heat exchange. These combined effects, together with the tectonic uplift of the circum-Arctic land masses^43^ all provide positive feedbacks for expansion of the Greenland Ice Seet (GIS).

In the modern context of increasing atmospheric greenhouse gas concentrations and rapidly declining Arctic sea ice, our study provides fundamental insights into consequences of a (seasonally) sea ice-free Arctic by demonstrating that limited sea ice presence in the Arctic Ocean and Iceland Sea together with a high Early Pliocene atmospheric CO_2_ concentration (380–400 ppm^17,18^) correspond to a strongly reduced GIS. The occasional presence of sea ice in the Early Pliocene Iceland Sea and Yermak Plateau was insufficient to provide an insolating buffer between the warm surface waters in the Nordic Seas and the GIS before ~4.0 Ma. Together with the high atmospheric Pliocene greenhouse gas concentrations, this setting likely inhibited major glaciation in Greenland. It is only when a more stable ice edge developed along the coast of East Greenland in the Late Pliocene^16^ and CO_2_ concentrations gradually decreased^17,18^ that the GIS could expand to reach the coast line^40,44^. As such, sea ice along the east Greenland coast acts as a positive feedback for sustaining and expanding the GIS. Our data do not allow disentangling the relative effects of Arctic sea ice extent and greenhouse gas concentrations on the GIS, which should be addressed in future modeling studies.

## Methods

### Age model

We studied the interval between 85.63–73.42 meter composite depth (mcd) of ODP Site 907. The age model is based on paleomagnetostratigraphy^45^. Ages for the three uppermost studied samples were calculated using the astronomically tuned IRD record from ref.^46^. All paleomagnetic reversals were updated to the most recent Astronomically Tuned Neogene Timescale 2012^47^ (Table 1). The alkenone SST reconstructions^29^ and the IRD record^38^ from ODP Site 907 (Fig. 2D,E) were placed on this age model to ensure a direct comparison. We adopted the age model reported in ref.^11^ for ODP Hole 910C and ref.^48^ for ODP Hole 911A.

**Table 1:** Early Pliocene tie points used for our age model.

ODP Hole	Depth (mbsf)	Depth (mcd)	Paleomagnetic reversal	Age model of Channell *et al*. (1999)	Update to Hilgen *et al*. (2012)
907A	66.8	74.54	Gauss/Mammoth	3.58	3.596
907A	70.5	78.24	Top Cochiti	4.18	4.187
907A	89.5	97.64	Base Gilbert	5.89	6.033

Depth (mbsf = meters below sea floor, mcd = meters composite depth) for polarity chron boundaries from ODP Hole 907A and the corresponding age from ref.^45^. We updated these ages to the Astronomically Tuned Neogene Timescale 2012^47^.

### Geochemical analyses

Biomarkers were analyzed at the Alfred Wegener Institute in Bremerhaven, Germany. Between 4 and 7 g freeze-dried and homogenized sediment was extracted using an accelerated solvent extractor (DIONEX, ASE200; 100 °C, 5 minutes, 1000 psi) with dichlormethane:methanol (2:1) as solvent. For quantification, internal standards, 7-hexylnonadecane, (7-HND; 0.076 µl/sample), cholesterol-d_6_ (10.5 µl/sample), and squalane (2.4 µl/sample), were added before analytical treatment. Separation of the extract into fractions hydrocarbon and sterol fraction was achieved via open-column chromatography with 5 ml *n*-hexane, and 6 ml *n*-hexane:ethylacetate (4:1 v/v), respectively with silica gel (SiO_2_) as stationary phase. The latter fraction was silylated with 200 µl BSTFA (bis-trimethylsilyl-trifluoroacet-amide) (60 °C, 2 h).

Compound analysis was performed by gas chromatography mass spectrometry (GC-MS). IP_25_ was analyzed using an Agilent 7890B GC coupled to an Agilent 5977A MSD using following heating program: 60 °C (3 min), 150 °C (rate: 15 °C/min), 320 °C (rate: 10 °C/min), 320 °C (15 min isothermal). Sterols (brassicasterol, 24-methylcholesta-5,22E-dien-3β-ol; campesterol, 24-methylcholest-5-en-3β-ol; β-sitosterol, 24-ethylcholest-5-en-3β-ol; dinosterol, 4α,23,24-trimethyl-5α-cholest-22E-en-3β-ol) were analyzed using an Agilent 6850 GC (30 m HP-5MS column, 0.25 mm inner diameter, 0.25 µm film thickness) coupled to an Agilent 5975C MSD (with 70 eV constant ionization potential, ion source temperature 230 °C). The GC oven was heated as follows: 60 °C (2 min), 150 °C (rate: 15 °C/min), 320 °C (rate: 3 °C/min), 320 °C (20 min isothermal). For both analyses, helium was used as carrier gas (1 ml/min constant gas flow). The injection volume was 1 µl (splitless). The identification and quantification of the IP_25_ monoene and sterols were performed by comparison of GC retention times with those of reference compounds and published mass spectra^49–51^.

### Palynology

Samples (10 cc) were dried, weighed and prepared using a standard palynological maceration procedure details in^52^ and for additional samples^27^ involving cold acid digestion (HCl, HF) of the mineral fraction, but no oxidation (full details in refs^27,52^). One *Lycopodium clavatum* tablet (batch #483216) was added prior to acid treatment. Occasionally mild ultrasonic treatment was applied before sieving at 10 µm and before mounting the organic residue (including dinoflagellate cysts, acritarchs) on microscope slides. Slides were then counted using a light microscope at x400 magnification along non-overlapping traverses until at least 250 specimens were counted, or until the slide was completely scanned.

## Supplementary information

Supplementary Figures

## Data Availability

All data generated or analyzed within this study are available at doi.pangaea.de/10.1594/PANGAEA.896652.
