# Supplementary material for: On the causes of Arctic sea ice in the warm Early Pliocene
Source: Sci Rep. 2019 Jan 30;9:989. doi: 10.1038/s41598-018-37047-y (PMC6353896; doi:10.1038/s41598-018-37047-y)
Supplement: Supplementary file 1 — Supplementary Figures [file 41598_2018_37047_MOESM1_ESM.docx]

**On the causes of Arctic sea ice in the warm Early Pliocene**

Caroline Clotten^1^*, Ruediger Stein^2,3^, Kirsten Fahl^2^, Michael Schreck^4^, Bjørg Risebrobakken^5^ and Stijn De Schepper^5^

**Affiliations**

^1^Uni Research Climate, Bjerknes Centre for Climate Research, Jahnebakken 5, 5007 Bergen, Norway.

^2^ Alfred Wegener Institute Helmholtz Centre for Polar and Marine Research, Am Alten Hafen 26, 27568 Bremerhaven, Germany.

^3^ MARUM and Faculty of Geosciences, University of Bremen, PO Box 330440, 28334 Bremen, Germany.

^4^Department of Geosciences, UiT The Arctic University of Norway in Tromsø, P.O. Box 6050, Langnes, 9037 Tromsø, Norway.

^5^NORCE Norwegian Research Centre, Bjerknes Centre for Climate Research, Bergen, Norway

***Corresponding author**: caroline.clotten @gmx.de

**Supplementary Figures**

**Supplementary Figure 1: IP_25_ vs. brassicasterol concentrations indicative for sea ice conditions.** Based on this plot, a classification of sea ice conditions into permanent sea ice, extended sea ice, seasonal sea ice (ice edge), and sea ice-free is possible. Zero or near-zero concentrations of IP_25_ and brassicasterol characterize permanent or perennial sea ice conditions. Ages of samples recording a “permanent” sea ice cover are indicated at (A) ODP Site 907 and (B) ODP Hole 911A (black) and ODP Hole 910C (green). Please note that there is a concentration difference between the Iceland Sea Site (A) and the Yermak Plateau Sites (B).

**Supplementary Figure 2: Variable modern sea ice extent between 1981 and 2017.** Colored lines indicate the March sea ice extent for the respective year and the mean from 1981 to 2010 (thick black line) in the Nordic Seas and sub-Arctic Ocean. Data is based on satellite data from http://nsidc.org/data/G02135^1^.

**Reference**

1 Fetterer, F., K. Knowles, W. Meier, M. Savoie, and A. K. Windnagel. Sea Ice Index, Version 3. Boulder, Colorado USA. NSIDC: National Snow and Ice Data Center. https://doi.org/10.7265/N5K072F8. [07/09/2017].
